# Gut Phage Database: phage mining in the cave of wonders

**DOI:** 10.1038/s41392-021-00615-2

**Published:** 2021-05-17

**Authors:** Magdalena Unterer, Mohammadali Khan Mirzaei, Li Deng

**Affiliations:** Institute of Virology, Helmholtz Center Munich and Technical University of Munich, Neuherberg, Bavaria Germany

**Keywords:** Gastrointestinal diseases, Microbiology, Systems biology

The diversity of the viruses in the human gut remains mostly unexplored. To shed some light on this known unknown, Camarillo-Guerrero et al.^1^ developed the Gut Phage Database (GPD), which includes 142,809 non-redundant gut phages obtained from analyzing 28,060 shotgun metagenome datasets.

The human intestinal tract is one of the most diverse microhabitats known, harboring billions of microorganisms including bacteria, viruses, fungi and archaea.^2^ Bacteria and their viruses, called phages, are the most abundant microbes in the human gut in an approximate ratio of 1:1 and a total abundance of about 10e13.^1–3^ Yet, we currently have little more than anecdotal data about gut phages relative to what we know about their bacterial hosts.^2^ The gut bacteria play central roles in human metabolism, immune modulation, and protection against pathogens.^2,4^ In addition, imbalances in their community contribute to human diseases or conditions such as Inflammatory Bowel Disease (IBD), allergies, obesity, and more.^2,3^ Similarly, changes in phage composition have been observed in IBD, type 2 diabetes, stunting, and Parkinson’s disease.^2,3^

Despite the renowned effects of phages on bacterial communities in other ecosystems, their function in the human gut is, for the most part, still unclear.^1–3^ This is due to the specific characteristics of phage genomes and the limited toolkit for studying them. Phage genomes are highly diverse, relatively small, and represent high levels of genetic mosaicism.^2,3^ They also lack universal gene markers unlike bacteria. Thus, their identification requires shotgun sequencing, which is prone to high background noise from human and bacterial genomes, which asks for extensive decontamination in the downstream analysis.^2,3^ Some experimental approaches like enriching VLPs before metagenome extractions can decrease contaminations by hosts’ genomes, but they also have some limitations.^5^ The public databases also do not sufficiently represent phage diversity resulting in most gut phages showing no significant homology to known reference sequences.^1–3^ Taken together, these challenges significantly increase the complexity of studying gut phages. As the result, earlier metagenomic studies found the majority of identified gut phages share no homology to public databases with a high variability between different studies, 14–87 %.^2,3^

**Fig. 1:**
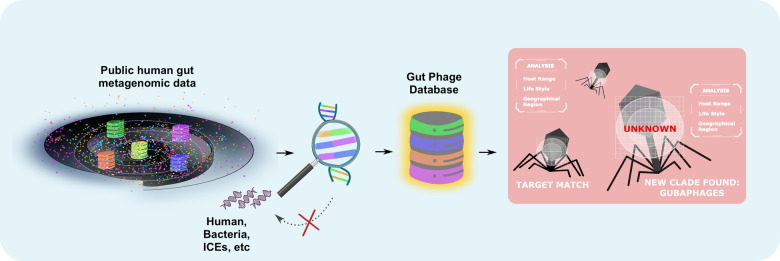
Schematic representation of key steps followed to develop the Gut Phage Database. The black halo hints at the viral dark matter. Databases, in colors, represent metagenomic data from different continents. The magnifier suggests the rigorous quality controls done. Phage scanning implies viral profiling and discovery. ICEs integrative and conjugative elements

To address the limitations with the public databases and expand our understanding of gut viral diversity Camarillo-Guerrero et al.^1^ developed the GPD which includes 142,809 non-redundant phage genomes obtained from mining 28,060 human gut metagenomes, and 2,898 genomes of cultured gut bacteria Fig. 1. Among phage genomes assembled, 9.4% were classified as complete, and 19.6% as high quality, as estimated by CheckV. The median phage genome size in GPD is ~31 kb, which is twice or three times longer than in other phage databases.^1^ For this, they used a rigorous quality control approach combined with an inhouse machine learning method to filter out the high background noise of metagenomic data and obtain complete viral genomes Fig. 1. Their machine learning approach uses gene density and k-mer frequency to distinguish and remove contamination with integrative and conjugative elements (ICEs). It also recognizes prophages that rarely enter the lytic cycle and mobile genetic elements. In addition, they predicted the host range of the GPD phages by screening them for CRISPR spacers, linking prophages to bacterial genomes, and then analyzing their co-occurrence with the predicted host to validate their predictions.^1^ Using this approach, 28.66% of phages could be assigned to 2157 strains of the host bacteria, while the highest diversity among phages linked to the Firmicutes phylum. In addition, about 36% of gut phages identified had broad host range and can potentially infect more than one bacterial species, which suggests that broad host range phages are more common in the human gut than previously hypothesized.^1^ Moreover, the epidemiological analysis of the GPD phages revealed 280 viral clusters (VCs) that are globally distributed over five different continents, at least. Phages that were found to be globally distributed had a broader host range compared to those seen only in one region. They could also see a clear separation of gut phages based on the geographical area they originated from and the human host lifestyles— living in rural vs. urban area.^1^ Finally, they discovered a new phage clade called Gut Bacteroidales phage or Gubaphage Fig. 1, which was found to be the second most prevalent VC in the human gut after the crAssphages— with no homology with these phages.^1^

Taken together, Camarillo-Guerrero et al. have significantly improved our understanding of the diversity, host range, and geographical distribution of unknown gut phages, as well as identified a new phage clade— highly common in the human gut. In addition, the high-quality, large-scale phage database of gut phages developed in this study will be a valuable resource for studying the role of phages in regulating human health. Yet, the full diversity of gut phages remains unexplored; no taxonomy could be assigned to the majority of reconstructed phages; the function of most phage proteins is still unknown, and diversity of RNA phages is not represented in the GPD database Fig. 1.
